# Improving Inhalation Performance with Particle Agglomeration via Combining Mechanical Dry Coating and Ultrasonic Vibration

**DOI:** 10.3390/pharmaceutics16010068

**Published:** 2023-12-31

**Authors:** Qingzhen Zhang, Zheng Wang, Kaiqi Shi, Hang Zhou, Xiaoyang Wei, Philip Hall

**Affiliations:** 1 Department of Chemical and Environmental Engineering, University of Nottingham Ningbo China, Ningbo 315100, China; qingzhen.zhang@nottingham.edu.cn (Q.Z.); zheng.wang@nottingham.edu.cn (Z.W.); 2 Key Laboratory for Carbonaceous Wastes Processing and Process Intensification Research of Zhejiang Province, University of Nottingham Ningbo China, Ningbo 315100, China; 3 Suzhou Inhal Pharma Co., Ltd., Suzhou 215000, China; skq@inhalpharma.com; 4 College of Pharmacy, Zhejiang University of Technology, Hangzhou 310014, China; 2552107179@zjut.edu.cn; 5 Nottingham Ningbo China Beacons of Excellence Research and Innovation Institute, University of Nottingham Ningbo China, Ningbo 315100, China; xiaoyang.wei@nottingham.edu.cn

**Keywords:** mechanical dry-coating, ultrasonic vibration, budesonide, magnesium stearate, inhalation

## Abstract

Agglomerate formulations for dry powder inhalation (DPI) formed with fine particles are versatile means for the highly efficient delivery of budesonide. However, uncontrolled agglomeration induces high deposition in the upper airway, causing local side effects due to high mechanical strength, worse deagglomeration, and poor fine-particle delivery. In the present study, fine lactose was mechanically dry-coated prior to particle agglomeration, and the agglomerates were then spheroidized via ultrasonic vibration to improve their aerosol performance. The results showed that the agglomerate produced with the surface-enriched hydrophobic magnesium stearate and ultrasonic vibration demonstrated improved aerosolization properties, benefiting from their lower mechanical strength, less interactive cohesive force, and improved fine powder dispersion behavior. After dispersion utilizing a Turbuhaler^®^ with a pharmaceutical cascade impactor test, a fine particle fraction (FPF) of 71.1 ± 1.3% and an artificial throat deposition of 19.3 ± 0.4% were achieved, suggesting the potential to improve the therapeutic outcomes of budesonide with less localized infections of the mouth and pharynx.

## 1. Introduction

Asthma is a chronic inflammatory disease associated with the expression of multiple inflammatory genes [1]. Inhaled corticosteroids (ICSs) are the most effective anti-inflammatory treatment for patients with persistent asthma. Inhaled budesonide is recommended for the treatment of asthma. However, the development of localized infections of the mouth and pharynx in clinical studies with Candida albicans occurs at an incidence of more than 5.5% when patients are treated with inhaled budesonide [2]. When such an infection develops, appropriate local or systemic (i.e., oral antifungal) therapy should be used if treatment with budesonide continues, or the therapy may need to be interrupted. Although patients are required to rinse their mouths after the inhalation of budesonide, there is no guarantee of the effect due to the low aqueous solubility of the drug. With the concerns regarding superior therapeutic outcome maintenance and the relief of local side effects, efficient fine particle delivery with reduced upper airway deposition for DPI budesonide formulations must be achieved [3].

To reach the deeper lung compartments, an aerodynamic diameter of 1–5 µm is required for respiratory particles [4,5]. Cohesive force dominates the behavior of micronized particles, leading to uncontrolled agglomeration and a wide size-distribution of the agglomerates [6]. The conventional approach to solve this problem in the pharmaceutical industry is blending micronized active compounds with coarse lactose [7]. Coarse lactose, working as an excipient, disperses the cohesive fine drug particles on the lactose’s surface to facilitate dispersion when inhaled and realizes dose metering during its manufacture. However, large proportions of lactose in a formulation increase the powder amount that must be inhaled due to the lower drug concentration in the formulation, which can result in cough and irritation. Both carrier-based (core–shell) and agglomerate formulations are widely and conventionally used in drug preparation. Various studies have been conducted to investigate techniques for the preparation of core–shell drug particles to improve drug administration. Engineering the surface properties to develop micronized particles with a core–shell structure [8,9,10] or to achieve particle agglomeration with cohesive force to realize the efficient aerosolization and flowability are effective approaches in drug delivery [11]. The core–shell-structured particles have an optical particle size that is larger than the aerodynamic particle size of 5 μm, but they exhibit lower inertia due to their lower density, which could contribute to its superior aerodynamics; in comparison, particle agglomeration utilizes the cohesion of fine particles to form an agglomerate, and they deagglomerate into fine particles via collision in the inhaler. The commercial products of carrier-based inhaler formulations usually have a fine particle fraction (FPF) in the range of 20–30% [12]. The use of a soft agglomerate formulation with less or without excipients is an alternative strategy to achieve high delivery efficiency [13]. Fine particle agglomeration works on the principle that fine particles form small-sized agglomerates due to their cohesive force, which become larger when fine particles adhere to the surface, layer-by-layer, during spheroidization in a coating pan when it rotates. These agglomerates are formed by a number of primary particles; they typically have diameters much larger than those of the original lactose particles. van der Waals forces dominate in agglomerates, along with capillary force, due to the linear decrease in van der Waals forces with the reduced particle radius [14]. In other words, the larger agglomerate particles consist of relatively smaller agglomerates, which are bonded to each other with the cohesive forces (usually van der Waals forces) of fine particles; therefore, there are more interaction points when the agglomerates become more compact as the weight of the agglomerates on the upper part press on their lower counterparts in the bottom of the rotation pan; therefore, the mechanical strength increases as spheroidization continues. Astra Zeneca’s Turbuhaler delivers formoterol in the microgram range through a soft agglomerate with an FPF of 40–50%, which is more than that of the carrier-based formulation. There were some improvements made to mitigate the side effects on the mouth or upper throat given its elevated deagglomeration and dispersion during inhalation. As soft agglomerates are produced by the conventional spheroidization process over a long duration to obtain good sphericity and flowability, their mechanical strength is normally quite high, which hinders the deagglomeration and fine particle dispersion performance [15]. The insufficient deagglomeration and breakdown of the agglomerates into fine particles during inhalation lead to improper penetration of the powder dosage to the bronchial area [16]. To address these issues, the conventional production of agglomerate formulation requires the following steps: (1) divide the commercial batch size into several sub-batches to minimize the impact of weight/pressure on the agglomerate particles in the bottom of the rotation pan; (2) sieve repeatedly to obtain a uniform size distribution of the agglomerate particles and remove of the larger agglomerate particles with higher mechanical strength [15].

**Figure 1:**
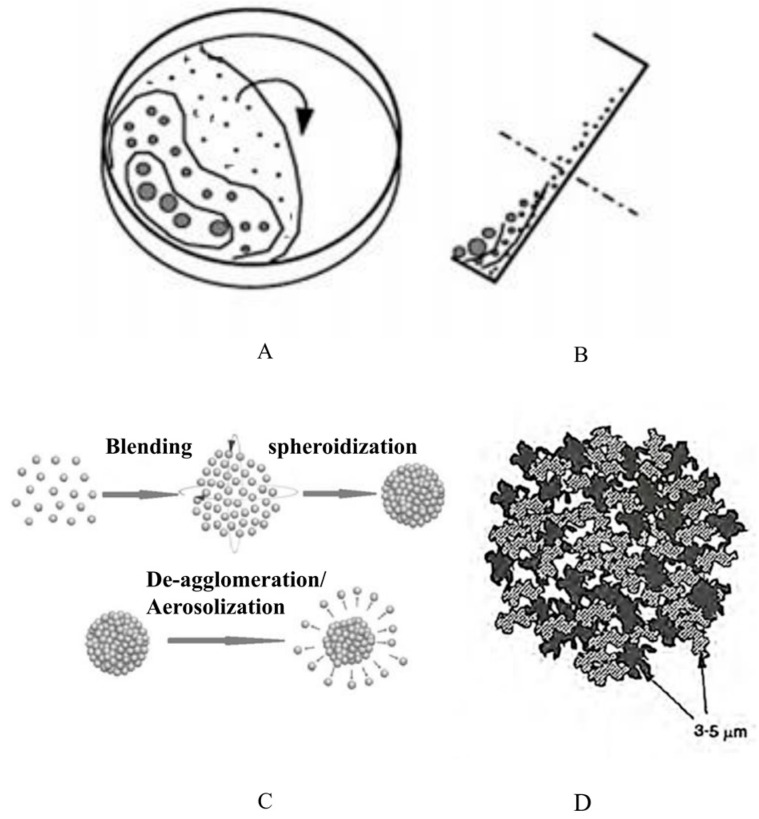
Schematic of (**A**) front view and (**B**) side view of particle agglomeration under ultrasonic vibration [20]. (**C**) Principle of particle agglomeration, and (**D**) schematic of individual agglomerate.

To solve the problems that occur during the conventional production of agglomerate formulations, achieving particle agglomeration under ultrasonic vibration is an alternative for the continuous production of agglomerate formulations with efficient deagglomeration and fine particle dispersion, which was originally reported for the collection of particulate matter of 2.5 μm or less in diameter [17]. Strong aerial ultrasonic waves induce the vibration of fine particles to generate interparticle collisions, resulting in particle agglomeration [18]. This is advantageous for producing agglomerates with loose microstructures that are ready for dispersion, because the spherical agglomerates are subject to continuous rotation during spheroidization, during which the agglomerates slide from the bottom of the coating pan (as shown in Figure 1A,B). The ultrasonic vibration waves are transferred to the agglomerates to induce small-amplitude vibration of the small aggregates to relax or even minimize the interaction points of the aggregates (as shown in Figure 1C,D). Therefore, finer particles and smaller aggregates with lower mechanical strength are present in the agglomerate formulation [19].

**Figure 2:**
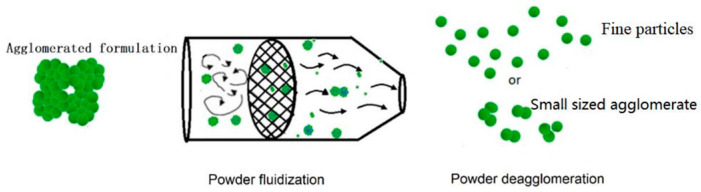
Schematic of pulmonary delivery processes of agglomerate formulation.

The pulmonary delivery process of agglomerate formulation is shown in Figure 2, which can be divided into four steps: (1) fluidization of DPIs within the inhaler device, (2) deagglomeration of DPIs, (3) transport of fine particles after dispersion through the oropharynx and throat, and (4) deposition of fine API particle in the lungs.

Agglomerates with higher mechanical strength reduce deagglomeration and result in small aggregates with larger inertia. The aggregates encase the API particles and carry them, which fall during the early impactor stages, resulting in a lower FPF. In contrast, smaller aggregates with lower mechanical strength deagglomerate and progressively break down into smaller fractions following collision with the interior wall of the device, and increase the fine particle detachment [21]. 

To further minimize the mechanical strength of agglomerate particles during spheroidization and achieve a uniform size distribution with superior flowability, interparticle cohesive forces should be decreased, and the surface energy of micronized powders during particle agglomeration could be lowered [22]. The mechanical strength of an agglomerate is proportional to the work of the cohesion of the particles, as previously described by Kendall and Stainton [23]. To reduce the intrinsic cohesion of fine powders, mechanical dry-coating techniques have attracted great interest for modifying the interparticle interactions in dry powders by changing their surface characteristics [24,25,26]. A single-step mechanical dry-coating method that is solvent-free should be simpler, cheaper, safer, easier to scale up, and more environmental friendly than liquid-based alternatives [27]. There are two conventional mechanical dry-coating systems with different blade geometries. The Nanocular^®^ system comprises a “press head”, which is configured to compress powders against the internal vessel wall. The Nobilta^®^ system is configured as a series of “propeller” blades that cause impact collisions within powders as the blades rotate [28]. Mechanical dry-coating creates intense shear and compression of the core (host) and coating (guest) particles via both impaction and compression as the particles are pushed between the edge of the press head and the chamber wall. The blade geometry, rotation speed, and gap from the wall can be tuned to facilitate coating but not size reduction, keeping heat generation or particle damage to a minimum [29]. This process breaks up the agglomerates of the cohesive host particles to expose their surfaces as the blades rotate at high speed. The reduction in interparticulate attractive forces from dry coating is through to occur by increasing the distance of the closest approach of the host particles or by reducing the contact area between two or more host particles. Given that the improvements in powder followability can be attributed to the reduction in intrinsic powder cohesion after coating for carrier lactose [30], mechanical dry-coating appears to be a technology that potentially improves the aerosol performance of high-dose DPI formulations by modifying fine lactose particles. 

Therefore, the objectives of the present work were, first, to assess the effect of ultrasonic agglomeration and mechanical dry-coating pretreatments on delivery efficiencies in terms of emitted dose (ED) and fine particle fraction (PFP) and, second, to investigate the synergic effect of ultrasonic and mechanical dry-coating on the dispersion of agglomerate formulations and the particle deposition on the upper airway. The combination of ultrasonic agglomeration and mechanical dry-coating is a promising approach to improve fine particle delivery efficiency while reducing the operation complexity and overcoming the drawbacks of conventional particle agglomeration methods. Moreover, the deagglomeration and in vitro dissolution performance of the formulation were also assessed.

## 2. Materials and Methods

The budesonide (purity: 98%) used in this study was purchased from Humanwell Healthcare (Group) Co., Ltd., (Yichang, China). Lactose (purity: 99%) was received as Inhalac 300 from DFE Pharma (Borculo, The Netherlands); the magnesium stearate (MgSt, purity: 99%) was acquired from PETER GREVEN (Venlo, The Netherlands), and all the materials used were of pharmaceutical grade.

### 2.1. Intensive Mechanical Dry-Coating

**Figure 3:**
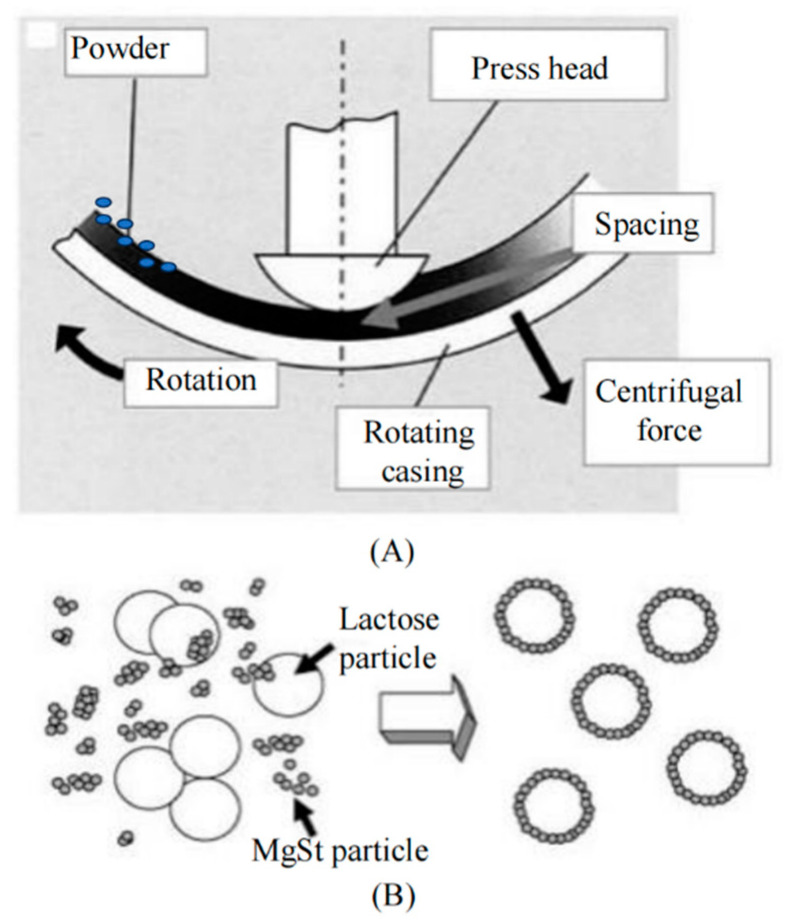
Schematic of mechanical dry-coating in a rotating device (**A**); guest particles coated and bonded onto the surfaces of host particles (**B**). Adapted from [32].

Mechanical dry-coating of drug powders with MgSt was carried out in a mortar grinder (MG100, Beijing Grinder Instrument Co., Ltd., Beijing, China). The grinder consisted of a rotating cylindrical chamber and a press head similar to the Nanocular^®^ system(Klausen Process Machinery, Kings Park NSW, Australia). The rotation speed varied from 100 rpm to 2000 rpm to create intense shear and compression, as illustrated in Figure 3. 

Due to the hydrophobic nature of budesonide, only lactose was dry-coated with MgSt to reduce the cohesive force. Each lactose sample (approximately 10 g) was blended with the tertiary component MgSt (0%, 0.5%, 1%, and 3%, *w*/*w*, labeled as M1, M2, M3, and M4, respectively) in a Cyclomixer (IM-0.1/1, HOSOKAWA MICRO B.V, Doetinchem, The Netherlands) at 300 rpm for 10 min according to preliminary study before being transferred to the cylindrical chamber of the grinder. The coating process was performed for 30 min at 120 rpm to coat the MgSt onto the host lactose particles [31].

### 2.2. Agglomerate Preparation via Ultrasonic Vibration

Budesonide was firstly micronized with an air jet mill (Aljet, DEC, Balerna, Switzerland) with micronization and a feed pressure of 8 bar to obtain a D_90_ value of less than 5 µm. The lactose mixture was then blended with micronized budesonide (4:1, *w*/*w*) in a cyclomixer at a mixing speed of 300 rpm for 30 min. The obtained mixture was then preagglomerated with a vibration feeder (BF-09, Suzhou Huilide Machine Co., Ltd., Suzhou, China) before being transferred to the rotary drum agglomerator (Wenling Aoli Machinery Co., Ltd., Wenling, China). The power supply of the vibration feeder was set at 160 V and 80 Hz with a variable-frequency digital controller (SDVC31-M, Nanjing CUH Science & Technology Co., Ltd., Nanjing, China).

**Figure 4:**
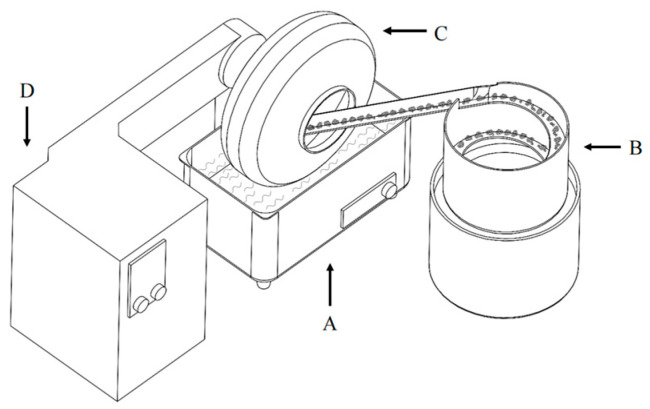
Schematic of the agglomeration apparatus, where A represents the ultrasonic cleaner, B is the vibration bowl feeder, and C and D represent the drum agglomerator and controller unit, respectively.

The agglomerates were smoothed and strengthened via sliding in a 30 cm diameter rotary drum agglomerator at 60 rpm for 10 min with its bottom immersed in an ultrasonic cleaner (Shenzhen Yujie Cleaning Equipment Co., Ltd., Shenzhen, China), as shown in Figure 4. The spherical agglomerates were produced at predetermined power inputs of 0 W, 100 W, 200 W, and 400 W, separately.

### 2.3. Analytical Methods

#### 2.3.1. API and Lactose Quantification

A high-performance liquid chromatography (HPLC) Arc™system (Waters Corp., Milford, CT, USA) with a C18 column (Waters SPHERISORB 3 μm, ODS 4.6 × 250 mm, Waters Corp., Milford, USA) was used for API quantification. The eluent was an acetonitrile (J&K Scientific. Beijing, China):water solution (40:60 *v*/*v*). The flow rate was set at 1.0 mL/min, and the injection volume of 20 µL. An ultraviolet (UV) detector (Waters Corp., Milford, CT, USA) was set at a wavelength of 240 nm. 

For lactose quantification, a C18 column (Agilent ZORBAX NH_2_ 5 μm 4.6 × 250 mm, 1634 Taguig, Philippines) and a refractive index detector (RID) (Type 2414, Waters Corp., Milford, CT, USA) were used. The mobile phase consisted of acetonitrile and water in a 70:30 (*v*/*v*) ratio at a flow rate of 1.5 mL/min. Both the column and RID were maintained at 30 °C during measurement. 

All the measurements were performed in triplicate.

#### 2.3.2. Particle Size Distribution

The particle size distribution of the materials before and after size reduction was determined with a HELOS laser diffractometer (Sympatec GmbH, Clausthal-Zellerfeld, Germany). Before measurement, the dispersion pressure was set at 3.5 bar. An R2 lens (measuring range 0.45–87.5 µm) was utilized with an optical concentration of 0.5–5%. The obtained data were analyzed with PAQXOS^®^ software (version 5.0, Sympatec GmbH, Clausthal-Zellerfeld, Germany). The particle size distribution is expressed as D_10_, D_50_, and D_90_ with mean and standard deviation (SD) from three measurements. 

#### 2.3.3. Density Properties of Powder Mixture

A agglomerate model can be built at the macroscopic level using surface adhesive attractions [23]. The fine particles make contact with each other at the molecular, level which causes adhesion, eventually forming an agglomerate of several hundred particles. Such an agglomerate displays porosity, elasticity, and strength that can be anticipated on a micrometer-scale [33]. As adhesion increases, agglomeration should increase [34], and the strength of adhesion decreases linearly with particle size. If the agglomerate is tight and condensed, the strength of an agglomerate can be surprisingly high if the particle size is small enough. However, there are always flaws and cracks in the composite structure of the agglomerate, which reduce its strength.

The deagglomeration behavior of an agglomerate formulation can be characterized using the mechanical strength (σ) of the agglomerate particle, which is calculated according to Equation (1) [35].

(1) $$\sigma =15.6\;(\frac{P_{f}^{4}w}{d})$$

The packing fraction $\left(P_{f}\right)$ is expressed as the ratio between the tap density $\left(\rho_{t}\right)$ and the true density of a powder ($\rho_{true}$), d is the diameter of particle, and w is the work of cohesion. 

According to the *Handbook of Pharmaceutical Excipients*, the true density of lactose and budesonide are 1.545 g/cm^3^ and 1.271 g/cm^3^, respectively [36]. Since the mass ratio of lactose and budesonide was fixed in the mixture, the packing fraction was proportional to the tapped density. The bulk density of powder was calculated according to USP-NF-35 by gently weighing (Sartorius analytic balance A200S, Goettingen, Germany) 10 mL of powder in a cylinder; the obtained weight was divided by the volume to calculate the bulk density (g/cm^3^). The tapped density ($\rho_{t}$) was measured after 5 min taps of the powders in a 10 mL cylinder using an automatic tapper (Powder Tester^®^, PT-X, Hosokawa Micron Powder Machinery Co., Ltd. Shanghai, China). The tapper was operated at a tapping rate of 250 taps per minute. Three replicates were carried out for each powder sample.

#### 2.3.4. Assessment of Surface Energy (SE)

A Surface Energy Analyzer (SEA, Surface measurement Systems, London, UK) and an inverse gas chromatograph (IGC) were used to assess the specific surface area (SSA) and the surface energy (SE), as previously reported [37]. A 150 ± 15 mg sample was weighed into presilanized glass columns (4 mm inner diameter × 30 mm length) with minimal tapping to avoid consolidation. Both ends of the column were loosely filled with presilanized glass wool to avoid powder movement. Prior to measurements, preconditioning of the column was conducted with 0% RH and 10 cm^3^/min nitrogen carrier gas flow for 60 min. 

Surface energy consists of a dispersive component and a specific Lewis acid–base component. An alkane series of octane to undecane served as nonpolar probes to calculate the dispersive component; chloroform and ethyl acetate were used as polar probes to predict the Lewis acid–base component [38]. The surface energy was measured at a surface coverage of 0.05 $p/p_{0}$ (where p is the partial pressure, and $p_{0}$ is the saturation vapor pressure). The measurement was conducted at 0% RH and 30 °C with a nitrogen gas flow of 10 cm^3^/min. The raw data processing was conducted using SEA Analysis Software Version 1.2.4.0 (Surface Measurement Systems Ltd., London, UK). SE calculation was based on the Dorris and Gray approach, as previously described [39]. Each powder sample was measured in triplicate.

#### 2.3.5. Agglomerate Size and Morphology Characterization

The dosing unit for the Turbuhaler^®^ is based on the volume principle and is constructed as a round disk with groups of conical holes placed at the bottom of the reservoir (as shown in Section 4). The agglomerates are forced into the dosing unit using a plastic scraper above the conical hole in a reproducible way when rotating the dosing wheel [40]. Therefore, size fractions of particles with different flowabilities should be present in the formulation to offset the voids during metering to ensure a lower variation in dose delivery [41].

The diameters and morphology of agglomerate particles were characterized using a digital optical microscope (ZML-310, Mengxin^®^, Shanghai, China) with a resolution of 3664 × 2748 pixels. A total of 50 particles were placed on a glass slide and sampled at 5× magnification in reflected light mode. Image analysis was conducted to determine the area-equivalent circle diameter ($X_{EQPC}$) and sphericity (defined as the ratio of the perimeter of an area-equivalent circle to the actual perimeter of the particle), following ISO 9276-6, with particle size analyzer softwareV5.30 (Mengxin^®^, Shanghai, China). 

Scanning electron microscopy (SEM, JCM-7000; JEOL^®^, Tokyo, Japan)–energy-dispersive X-ray analysis was applied to observe the agglomerates’ morphology and the elemental composition of MgSt in the mixture. The sample was fixed on the carbon tape and a Smart Coater (DII-29030SCTR) was used with an electrical current of 3 A for 2 min to conduct the gold sputter-coating at 0.67 Pa with a working distance of 12.5 mm before the measurement. For imaging, an acceleration voltage of 15 kV was used. 

#### 2.3.6. Aerodynamic Assessment

The aerodynamic performance of the agglomerate formulation was characterized with a Next-Generation Impactor (NGI). A flow rate of 60.0 L/min (±5%) was set, with a pressure drop of 4 kPa provided using a vacuum pump (HCP 5 Copley Scientific Ltd., Nottingham, UK) to allow 4 L of air through the inhaler within 4 s in each measurement. The flow rate and the suction time were controlled with a flow meter (DFM2, Copley Scientific, Nottingham, UK). 

The spherical agglomerates of about 150 ± 15 mg were initially weighed and filled into the Turbuhaler^®^ (the capacity of the dose chamber was 1 mg per action). The budesonide and lactose deposited in the mouthpiece and different NGI stages were rinsed with an eluent phase and characterized with API and lactose quantification methods. To avoid particle bouncing and shifting to the later NGI stages during deposition, 0.5% (*w*/*v*) silicone oil in n-hexane solution was used to precoat the impactor stages before measurement. The emitted dose (ED), fine particle fraction (FPF), and fine particle dose (FPD) were calculated from the measured content of budesonide for each stage. PD and FPF are the mass and percentage of the drug with an aerodynamic diameter of between 0.5 and 5 μm, respectively, calculated as the ratio to the ED.

#### 2.3.7. Dissolution of Aerosolized Particles

**Figure 5:**
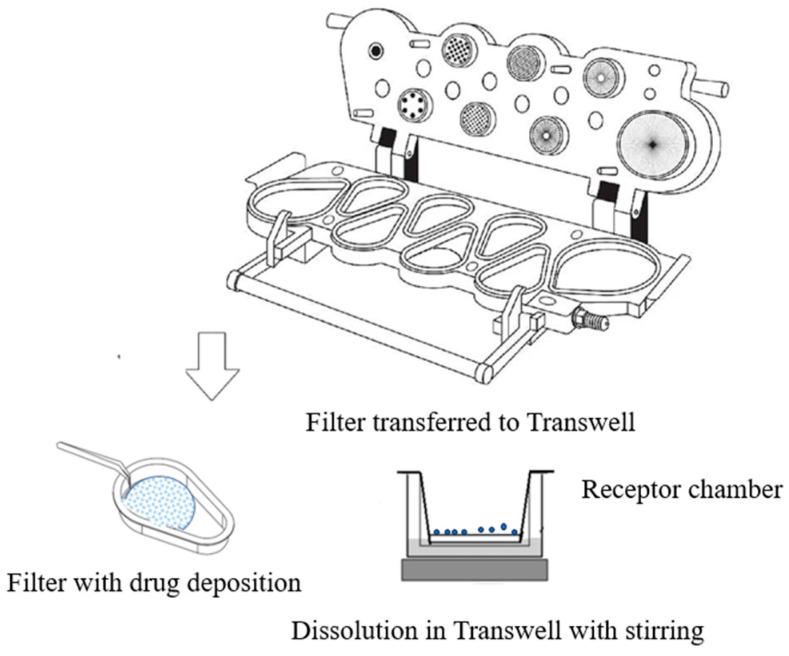
Schematic of the in vitro dissolution of aerosolized particles.

The aerosolized particles from the Turbuhaler^®^ deposited during stage 4 of NGI were collected with an insert Regenerated Cellulose Membrane Filter (RC55, 4.7 cm diameter, and 0.45 µm, Whatman^®^ Corporation, Shrewsbury, MA, USA) on the impactor stage at 60 L/min. The amount of powder collected was estimated based on the previous NGI experiment. After aerosolization, particles deposited on the filter were then obtained from the NGI stage for the dissolution test. A total of 20 mL of the dissolution medium (0.5% *v*/*v* tween-80 solution), prewarmed at 37 ± 0.2 °C with a transdermal diffusion cell system (Tianjin Shengda Sanhe Optical Instrument Co., Ltd, Tianjin, China), was filled into the receptor chamber and magnetically stirred; a glass fiber filter was inserted onto the bottom of the Transwell and fixed with a metal clip to avoid medium leakage. The dissolution started with the glass fiber filter acting as a diffusion membrane in contact with the medium, as shown in Figure 5. 

At each predetermined sample time (0, 10, 20, 30, 45, 60, 90, 120 min), 0.5 mL of the dissolution medium was collected, and then the same volume of prewarmed medium was replenished in the receptor chamber. Three parallel experiments were conducted, and the results are expressed as a percentage of the drug dissolved at a certain time point compared to the total drug dissolved. 

#### 2.3.8. Mechanical Strength Measurement

The mechanical strength of a single soft agglomerate was determined following a previously reported micromanipulation method [42]. Before measurement, the force transducer was calibrated according to the force calibration protocol recommended by Yan [43]. The agglomerate particles were sieved to obtain a size fraction between 355 µm and 500 µm. Twenty agglomerate particles in this size range were randomly selected as representative sample particles. The sample particle was placed on a glass plate and moved below the force probe with the assistance of a view camera. After that, the force probe that was connected to a force transducer (Model 406A, Aurora Scientific Inc., Aurora, ON, Canada) compressed the soft agglomerate at a preset speed of 1 µm/s until the agglomerate ruptured. The voltage signal from the force transducer was recorded with a data acquisition apparatus (PC-100, NARISHIGE Science Instrument LAB, Tokyo, Japan) to calculate the rupture force. The deformation at rupture (the diametric compressive displacement when the rupture occurred) was determined from the voltage versus time curve directly. The obtained data were analyzed with Micro-Particle Strength Analysis Software (v2.1.x, Micromanipulation, and Microencapsulation Research Group (µCAP)). The measurements were performed at a controlled temperature (20 ± 2 °C). 

#### 2.3.9. Deagglomeration and Dispersion Behavior Characterization

Deagglomeration occurred when agglomerates were exposed to impaction or shear force induced via airflow through an inhaler device. To investigate the effect of ultrasonic vibration on deagglomeration, the assessment was conducted using dry dispersion laser diffraction, as previously reported [44]. Briefly, 5 mg of soft agglomerate was loaded in a Sympatec HELOS/RODOS laser diffractometer testing tube with an R2 lens (0.45–87.5 µm), and we manually set the dispersion pressure (DP) from 0.2 to 4.0 bar for particle size measurement, which would allow reliable assessment of the agglomerates’ dispersibility without inhaler device effects. The measurement started when the optical concentration ($C_{opt}$) exceeded 0.5% and stopped when the $C_{opt}$ fell below 0.3%. The measurements at each dispersion pressure were conducted in triplicate. 

The degree of deagglomeration (DA) at each dispersion pressure was calculated with Equation (2).

(2) $$\mathrm{DA}=\frac{\mathrm{DH}}{D_{x}}$$

$D_{x}$ is the median particle size (D_50_) at each dispersion pressure (0.2–4.0 bar), and DH is expressed as the D_50_ value measured at 4 bar (full deagglomeration). The dispersion pressure could break the agglomerate particles into fine particles, and subaggregate, $D_{x}$ revealed the extent of deagglomeration, with a lower value indicating better deagglomeration. DA was plotted against a series of dispersion pressures to represent the deagglomeration behavior of the agglomerate formulation.

To further investigate the effect of mechanical dry-coating in combination with ultrasonic agglomeration on fine particle dispersion using a Turbuhaler^®^, a modified modular Sympatec HELOS was used for real-time monitoring of the fine-particle release profiles. An inhaler device adapter, artificial throat, and preseparator were separately integrated with the Sympatec HELOS. Th artificial throat and preseparator were used to simulate the human throat and bronchi bifurcation, where the detachment processes of fine particles occurs. Particles with larger inertia impacted the artificial throat and were captured. Fine particles with smaller inertia could successfully pass through the artificial throat and were determined using a laser detector. 

The measurements were conducted under the following conditions:

1: The start and stop of the measurements with an R2 lens were triggered at $C_{opt}$ values of 1.0% and 0.5%, respectively; 

2: The measurement duration was 4 s with the data recorded in 100 ms time intervals at 60 L/min. 

Each sample was quantified in triplicate. PAQXOS^®^ software (Sympatec GmbH, Clausthal-Zellerfeld, Germany, version 5.0) was employed to analyze the data. The released amount of particles (R) recorded in each 100 ms was calculated according to Equations (3) and (4)

(3) $$R=C_{\mathrm{opt}}\times {\mathrm{dQ}}_{3}$$

where ${\mathrm{dQ}}_{3}$ represent the volume percentage of particles within a size range (%).

(4) $${\mathrm{dQ}}_{3}=Q_{3}\left(D_{i1}\right)-Q_{3}\left(D_{i2}\right)$$

$Q_{3}\left(D_{i1}\right)$ represents the ratio of the total volume of particles that are smaller than $D_{i\;}$ to the total volume of all particles. $D_{i}$ (µm) represents the instantaneous particle size measurement of each 100 ms within 4 s duration. The particle fraction between D_10_ of budesonide ($D_{i2}$ = 0.5 µm) and D_90_ of lactose ($D_{i1}$ = 7 µm) was captured during measurement. The release amount profile was plotted with R versus time (t) using Origin 2023b Software (Origin Lab, Northampton, MA, USA). 

### 2.4. Statistical Analysis

Every experiment was conducted in triplicate (or more). For statistical analysis comparing two groups, Student’s *t*-test was applied with SPSS software (IBM SPSS Statistics, V 28.0). The probability values that were <0.05 (*p* < 0.05) were considered as statistically significantly different.

## 3. Results

### 3.1. Bulk Properties of Powder Mixture

As shown in Table 1, the API particles were milled to an inhalable size range, with a D_90_ of 3.23 ± 0.15 µm. To facilitate mechanical dry-coating, sample materials were selected so that lactose (LH300) had a larger D_90_ of 6.97 ± 0.62 µm than MgSt (3.07 ± 0.05 µm). The D_90_ showed negligible changes for different concentrations of MgSt, indicating the minimal influence of mechanical dry-coating on particle size. The surface area decreased after mechanical dry-coating compared to the original state, which revealed that the high surface energy sites were gradually occupied by the MgSt particles.

**Figure 6:**
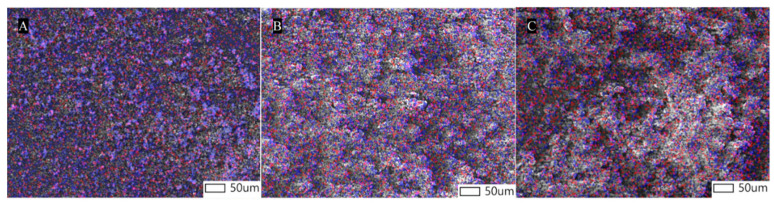
SEM-EDX images of mechanical dry-coating with different concentrations of magnesium stearate: (**A**) 0.5%, (**B**) 1%, and (**C**) 3%. Red represents magnesium and blue represents oxygen.

The characterization of the mechanical dry-coating uniformity was performed using SEM-EDX (energy-dispersive X-ray analysis), as depicted in Figure 6. The measured molar fraction of the magnesium in the mixture surface was in fair agreement with that calculated via stoichiometry, as shown in Table 2, indicating a nonsignificant loss of MgSt during the coating process.

The bulk properties of the mixture after mixing with budesonide were measured and are presented in Table 3. All the formulations had a lower SD (<2%) in terms of drug content and can be considered excellent and suitable inhalable blends [45]. The tapped density of the mixtures increased as powders were mechanically dry coated but did not show any further increase with increasing MgSt concentration. However, the surface energy decreased significantly to 99.3 ± 2.1 mJ/m^2^ when 3% MgSt was incorporated, which can be compared to the 315.0 ± 8.0 mJ/m^2^ without mechanical dry-coating. Therefore, although the increase in the packing fraction could be related to an exponential increase in the mechanical strength of powder agglomerate [35], the substantial reductions in powder surface energy offset this increasing trend.

### 3.2. Production of Binary Agglomerate Formulation with Ultrasonic Vibration

#### 3.2.1. Bulk Properties of Agglomerate Formulation

**Figure 7:**
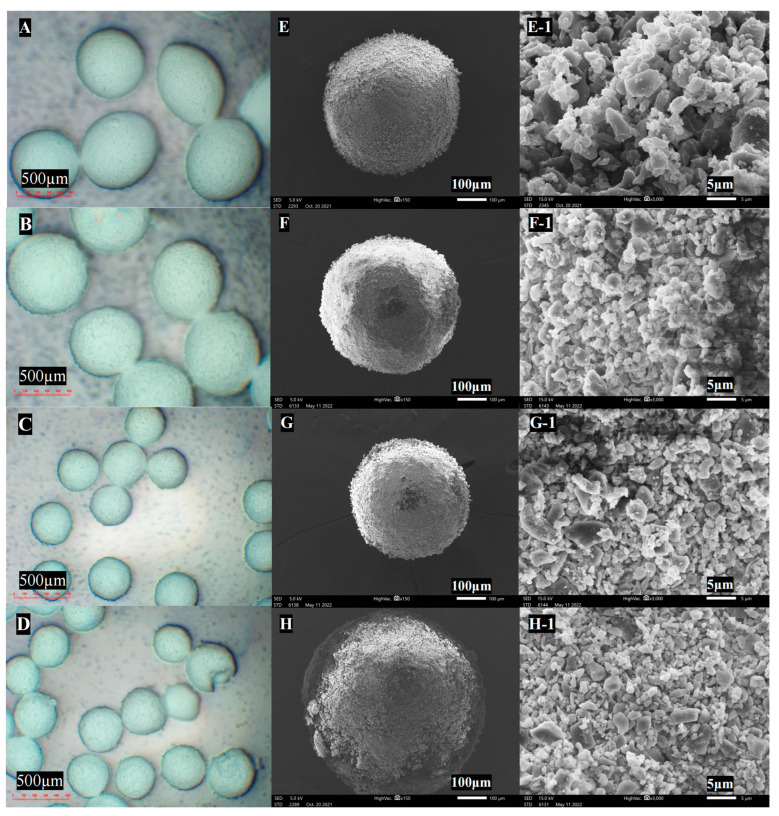
Optical microscope images of agglomerate under ultrasonic vibration: (**A**) 0 W, (**B**) 100 W, (**C**): 200 W, and (**D**) 400 W; SEM images of agglomerates and corresponding surface: (**E**,**E-1**) 0 W, (**F**,**F-1**) 100 W, (**G**,**G-1**) 200 W, and (**H**,**H-1**) 400 W (magnification of microscope for agglomerate: 50×; SEM for agglomerate: 150×, for surface: 3000×).

Ultrasonic vibration contributed to a smaller agglomerate size and higher sphericity, as observed with an optical microscope, as shown in Figure 5 and Table 4. Further observation with SEM revealed structural differences in the agglomerate surface. Ultrasonic vibration contributed to loosening structure, with individual micro-sized particles evenly packed together, indicating a better dispersion of the fine particles. However, for the formulation that was not subject to ultrasonic vibration, the agglomerate surfaces were covered with clumped and interlocked particle aggregates, as shown in Figure 7F1–H1. Ultrasonic vibration also improved the drug content in the agglomerate formulation by decreasing the powder’s adherence.

The rupture force decreased gradually from 5.3 ± 0.5 mN to 0.8 ± 0.2 mN as the ultrasonic power increased from 0 W to 400 W. Moreover, the rupture deformation of agglomerates was within 10%, suggesting that these agglomerates could be considered elastic particles [46]. The similar rupture deformation of the agglomerate formulation for each vibration intensity suggested the minimal destruction of the agglomerate structure during the filling and packing in the manufacturing process. Although ultrasonic vibration decreased the mechanical strength, it produced agglomerate particles mechanically stable at 200 W, which is suitable for pharmaceutical manufacture, and was further validated by comparison with the rupture force determined from the agglomerate particles in a commercial Turbuhaler^®^ (rupture force: 1.69 ± 0.47 mN, *n* = 20). 

#### 3.2.2. In Vitro Aerosolization Performance

**Figure 8:**
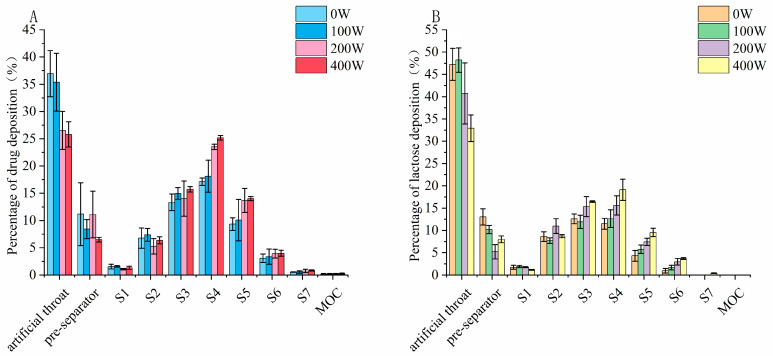
Aerodynamic deposition profile of (**A**) budesonide and (**B**) lactose under ultrasonic vibration.

The aerosolization performance of the formulation produced through ultrasonic vibration is listed in Table 5. There was a minor increase in the ED for both budesonide and lactose for power inputs from 0 W to 400 W; the FPF was improved from 36.2 ± 3.0% to 53.2 ± 4.1% for budesonide and from 31.8 ± 4.1% to 52.5 ± 4.6% for lactose. Further observation of the aerosol deposition profile shown in Figure 8 revealed the magnitude of the improvement in the deposition fraction on the NGI stages, which followed the order of stage 4 > stage 5 > stage 6 for budesonide and lactose. Reductions in the deposition on the artificial throat and preseparator could also be observed under ultrasonic vibration, indicating that the breaking up of agglomerate into fine inhalable particles was more efficient after ultrasonic vibration treatment for both components. 

### 3.3. Production of Ternary Formulation with Combination of Mechanical Coating and Ultrasonic Vibration

Because of the significant effect of the incorporation of MgSt on lowering the cohesive force of fine particles and enhancing the aerosol performance of DPI [47], the effect of mechanical dry-coating on its own and in combination with ultrasonic vibration (200 W power input) on the spherical agglomerate bulk properties and aerodynamic deposition was further investigated. As shown in Table 6, the combination of ultrasonic vibration and mechanical dry-coating contributed to an overall improved drug content but decreased mean $X_{EQPC}$ and rupture force compared to those achieved with mechanical dry-coating on its own. 

**Figure 9:**
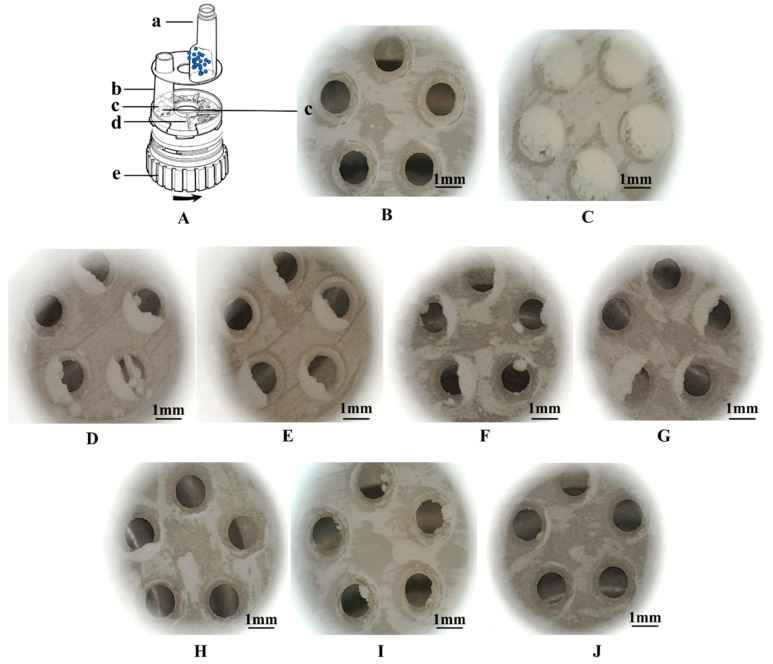
Schematic of Turbuhaler ^®^ in disassembled form (**A**): a. reservoir for agglomerate particles, blue dots represent the aggregate particles, b. inhalation channel, c. dosing unit, d. plastic scraper, e. dosing wheel [41]; images before (**B**) and after metering (**C**). (**D**–**G**) The residual in dosing unit after inhalation for ultrasonic power of 0 W to 400 W, respectively. (**H**–**J**) Mechanical dry-coating with 0.5%, 1%, and 3% MgSt, respectively.

In terms of aerodynamic properties, mechanical dry-coating contributed to a higher ED compared to ultrasonic vibration, demonstrated by the lower amount of residual aggregates in the metering hole, as shown in Figure 9. Further improvement was achieved when combined with ultrasonic vibration. On average, both approaches improved the FPF at elevated MgSt concentrations. Spheroidization with ultrasonic vibration resulted in an overall higher FPF and achieved a plateau of 71.1 ± 1.3% with 1% MgSt coating.

**Figure 10:**
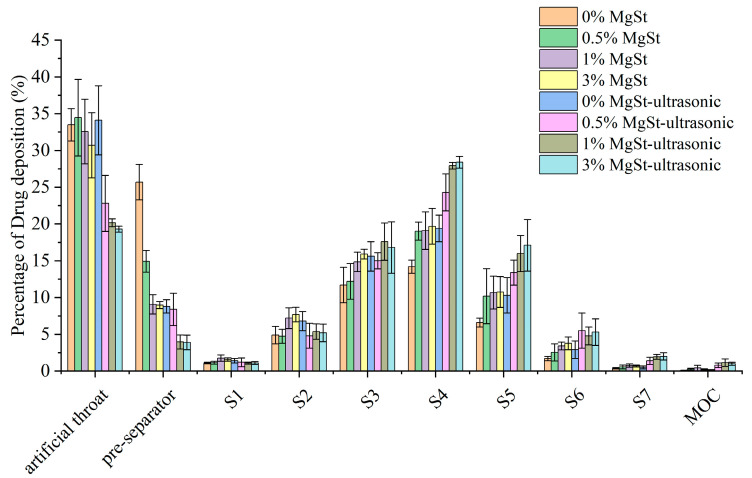
Aerodynamic particle size distribution of soft agglomerates with different concentrations of MgSt added to the formulation and subjected to spheroidization (*n* = 3).

To investigate the effect of the preparation method on the deagglomeration and dispersion in detail, the particle deposition fractions were further compared. Ss shown in Figure 10, there was a significant reduction in the deposition in both the artificial throat and preseparator with the combined method, but only a significant reduction was achieved in preseparator deposition with mechanical dry-coating alone. Moreover, the combined method contributed to more fine particle being deposited, especially from S4 to S7, which were used to characterize the deposition behavior in the lower region of the lung.

### 3.4. Deagglomeration and Dispersion Performance of Soft Agglomerates

**Figure 11:**
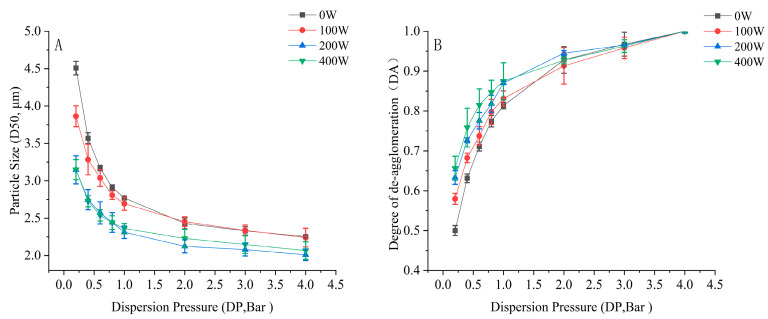
Deagglomeration performance of formulations with ultrasonic vibration: (**A**) particle size versus dispersion pressure curve and (**B**) deagglomeration profiles for agglomerates (*n* = 3).

The particle size–dispersion pressure curve and deagglomeration profiles differed, as shown in Figure 11, indicating distinct deagglomeration behaviors with different ultrasonic power inputs. Lower D_50_ values were observed for the formulation produced with an ultrasonic power of 0 W to 200 W at predetermined dispersion pressures. The further increase to 400 W failed to produce further effects. The DA versus dispersion pressure profile also illustrated a trend of higher relative deagglomeration being achieved as ultrasonic vibration power increased, indicating differences in the cohesive properties and mechanical strength of the formulations produced using different ultrasonic powers. At low dispersing pressures, e.g., 0.2 bar, the DA of the agglomerate formulation without ultrasonic vibration was low (DA = 0.50). A DA value of 0.66 was achieved with 400 W, indicating more powder remained agglomerated in the formulation without ultrasonic vibration when inhaled and demonstrating a lower FPF [44].

**Figure 12:**
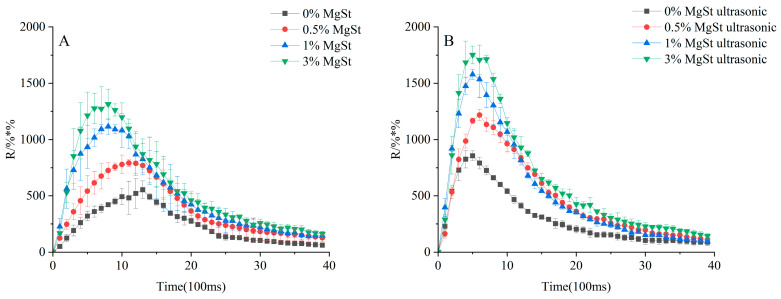
Release profile of formulations with mechanical dry-coating alone (**A**) and in combination with ultrasonic vibration (**B**). R_max_ represents the maximum release amount at T_max_, T_max_ represents the time to R_max_, and R_AUC_ is the total release amount.

Further investigation of the fine particle dispersion was performed to determine the effect of mechanical dry-coating alone and in combination with ultrasonic vibration on the fluidization and dispersion processes. The release profile of the DPIs is depicted in Figure 12, where the amount of fine particles released presents a trend of first increasing and then decreasing with increasing MgSt concentration in both scenarios. Specifically, the DPIs with 0.5% and 1% showed approximately a 1.5- and 2-fold increases in R_max_ compared with that of 0%, respectively. However, there was no significant difference in the R_AUC_ between 1% and 3% MgSt (*p* > 0.05). Overall, the formulation produced via the combination of ultrasonic vibration and mechanical dry-coating demonstrated had a shorter T_max_ and a higher R_AUC_ than that produced via mechanical dry-coating alone with the same MgSt concentration. These results showed that the improved fine particle dispersion of the agglomerate formulation was achieved by combining mechanical dry-coating with ultrasonic vibration, which produced readily deagglomerated particles to facilitate fast entrainment of the fine particles out of the inhaler via airflow. 

### 3.5. Dissolution Evaluation of Aerosolized Particles

**Figure 13:**
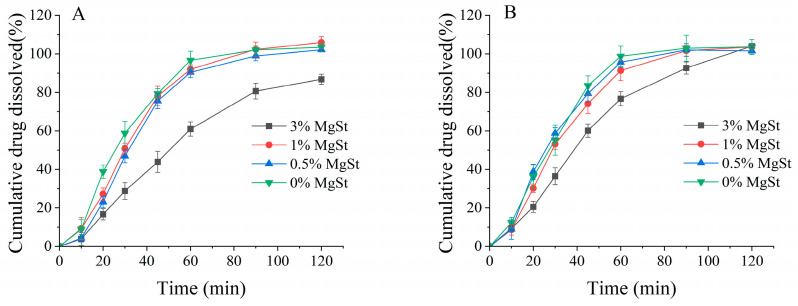
Dissolution of aerosolized drug particles from agglomerates obtained via mechanical dry-coating alone (**A**) and in combination with ultrasonic vibration (**B**) (*n* = 6).

Mechanical dry-coating of MgSt on DPI enhances fine particle deposition but influences the dissolution rate, as previously reported [47]. The dissolution of aerosolized drug particles on the surface of the lung epithelium is the first step in achieving good bioavailability. Figure 13 illustrates the overall similar dissolution trend for the formulations prepared with both processes. A relatively faster dissolution was revealed for the formulation produced via ultrasonic vibration. The dissolution profile for all formulations could achieve a plateau indicating full dissolution at 120 min; however, the formulations with 3% MgSt exhibited slower dissolution. For mechanical dry-coating, the incorporation of 3% MgSt resulted in an incomplete dissolution of approximately 85%, while complete dissolution could be observed when ultrasonic vibration was employed. 

## 4. Discussion

Spherical agglomerate formulations are initially spheroidized using a rotary drum agglomerator via the agglomeration of a micronized powder [48]. During the spheroidization process, the fine particles compact and collide, resulting in an uncontrollable size distribution and larger agglomerates, which deteriorate the metering in the Turbuhaler^®^ [41,48]. Therefore, a continuous production process is not feasible due to the repeated sieving process that is required to obtain the required size fraction. Moreover, the compaction of the formed agglomerates results from the self-weight during spheroidization, which also increases the mechanical strength of the agglomerate particles to impair deagglomeration and dispersion. More recent research reported a twin-screw extruder and a vibration chute with a frequency-controlled speaker connected to the end to achieve the continuous production of agglomerate particles [49]. However, the vibration was not able to be uniformly transferred to the chute from one end to the other, resulting in variable mechanical agglomerate strength and an inconsistent size distribution. The specific advancements achieved in this study include the combination of ultrasonic vibration and mechanical dry-coating for particle agglomeration and can be summarized as follows: (1) the continuous production of agglomerate particles with uniform size distribution was realized; (2) aerosolization performance was improved, benefitting from the lower mechanical strength of spherical agglomerates with better deagglomeration and reduced fine particle interaction forces; (3) aerosol performance was improved and upper airway deposition, caused by a larger fine particle fraction and lower artificial throat deposition, was reduced. 

Ultrasonic waves cause large-amplitude vibration in small particles and small-amplitude vibration in large particles [17]. The collisions between fine particles promote agglomerations with lower mechanical strength compared with conventional spheroidization [17], because spherical agglomerates are subject to the weight compression of the powders during spheroidization. When agglomerates slide upward from the bottom of the mixture, ultrasonic vibration is transferred to the agglomerates to induce small-amplitude vibration in small aggregates for the relaxation or even minimization of the interaction points of the aggregates. Therefore, larger amounts of fine particles and smaller aggregates are present in an agglomerate formulation when aerosolized. Traditional ultrasonic agglomeration devices combine a vibration apparatus with an ultrasonic wave generator via a metal connection, so ultrasonic waves cannot be uniformly transmitted to fine particles. Ultrasonic waves in a water bath avoid the vibration dead zones that occur during spheroidization: the obtained agglomerates are more uniform in size, with a sphericity over 0.9, sufficiently facilitating dose metering in the manufacturing process [13]. In this study, ultrasonic vibration improved the aerosol performance of agglomerate formulations by reducing the mechanical strength and conserving the elastic properties, characterized by rupture force and rupture deformation, respectively [50]. This can be explained by the relatively higher DA at lower dispersion pressure compared with that of formulations without ultrasonic vibration. The viscous shear stress across an inhaler could be considered approximately equivalent to a 0.1 bar dispersing pressure [51], which is sufficient for deagglomeration characterization at lower dispersion pressures, serving as an indicator of dispersibility [44]. By contrast, agglomerates prepared without ultrasonic vibration required higher dispersing force to overcome the interparticulate interactions, indicated by its lower FPF.

The improvement in aerosolization with ultrasonic vibration could be further illustrated by the lactose deposition on the impactor stage. A significant reduction in deposition of budesonide in the artificial throat and preseparator was observed when followed by ultrasonic vibration, as shown in Figure 6. Aggregates with larger inertia encased the API particles, which fell on the early impactor stages, resulting in lower FPF; the smaller aggregates produced via ultrasonic vibration deagglomerated and progressively broke up into smaller fractions following the collision with the interior wall of device, consequently increasing fine particle detachment [21]. Moreover, ultrasonic vibration contributed to the improvement in fine lactose deposition, which was related to the decreased rupture force liberating more fine particles during deagglomeration. Fine drug particles existed either as individual particles or attach to fine lactose particles to form drug–lactose agglomerates, which were delivered to the impactor stage, resulting in codeposition, which enhanced fine particle deposition, as previously reported [52]. 

The mechanical strength of an agglomerate is proportional to the work of the cohesion of the particles, as previously described by Kendall and Stainton [23]. To reduce the intrinsic cohesion of the fine powder [24,25,26], the mortar grinder used in this study had a press-on force to create frictional force to ensure the uniform coating of the powder mixture by changing their surface characteristics [22]. The IGC results confirmed that the intrinsic interparticle cohesion of the mixture could be substantially reduced after mechanical dry-coating, because the surface energy of the micronized budesonide determined using IGC is 68 mJ/m^2,^ according to the previous literature [53]. Additionally, the fine lactose contributed the majority of the cohesion of the mixture. The real-time monitoring of fine particle release also demonstrated improved fine particle release with elevated MgSt concentration, which correlated well with their corresponding FPF and rupture force. Moreover, the significant increase in ED after mechanical dry-coating attributed to the reduction in the interparticle interactions of the powder, resulting in less powder residuals in the metering hole after inhalation compared with that of the binary formulation without MgSt. 

The dispersion depended on the drag force on the particle from the airflow, the interaction force between particles, and the collisions between the particles and spiral channel when DPI particles were released from the Turbuhaler^®^ [54]. For the mechanical dry-coating process, although the rupture force of the agglomerated particles was reduced to improve dispersion, the mechanical strength was maintained at about 4 mN and failed to decrease with increasing MgSt concentration. Therefore, the aggregates that failed to deagglomerate completely within a short time period were prone to remaining in the metering hole or inhalation channel and gradually eroded until the release was complete. Larger particles were deposited in the artificial throat or preseparator stages: the R_AUC_ and R_max_ were low, while T_max_ was long. These results suggested that a higher mechanical strength of the agglomerate particles led to a gradual release of fine particles, resulting in an early deposition of DPIs in the oropharynx instead of being delivered to the lower airways. However, combining mechanical dry-coating with ultrasonic vibration had a synergistic effect on both decreasing the mechanical strength and reducing the cohesive force within fine particles to achieve the bursting inhalation and release of high-concentration aerosol clouds in short intervals, resulting in an increased R_max_ and a shortened T_max_. Given the lower mechanical strength and interaction force, the pressure drop during inhalation provided sufficient kinetic energy, achieving a higher R_AUC_ and efficient pulmonary drug delivery. The result was also in accordance with the corresponding FPF. 

A dissolution study of aerosolized particles on NGI stages was conducted to investigate the diffusion capacity of aerosolized drug particles across a permeable membrane; the artificial membrane acted as an air–liquid interface to mimic the lung’s surface [55,56]. The literature suggests that drug powders coground with MgSt could slow the wetting process and decrease the dissolution rate due to their hydrophobic properties [57]. In this study, the dissolution results revealed that only mechanical dry-coating with 3% MgSt could substantially reduce the dissolution rate. It was also interesting to note that despite the apparent dissolution decreases occurring with higher MgSt concentrations, combining mechanical dry-coating with ultrasonic vibration contributed to a complete dissolution at a 120 min time point and demonstrated an overall faster dissolution rate compared to that achieved with mechanical dry-coating only. These contrary observations could be caused by the better dispersion of the fine budesonide particles with lactose via ultrasonic vibration, which increased the wettability of the API. These results indicated that ultrasonic vibration could better disperse agglomerates and allow for increased fine particle deposition on the diffusion membranes; therefore, the powder wetted faster when contacting the medium, as previously reported in the literature [58,59]. This comprehensive factor indicated the sufficient dispersion of API, which enhanced the solubility and hydrophobicity of MgSt to hinder dissolving. This was only true with lower MgSt concentrations, because the ultra-thin layer of the MgSt coating on the lactose surface may not have been sufficient to hinder water penetration and decrease the dissolution rate, but once the hydrophobic component was increased to a certain limit, slower dissolution rate was observed [47]. For the 3% MgSt formulation, it appeared that drug particles were encased in lactose aggregates and hindered the wettability, resulting in slower dissolution, but ultrasonic vibration dispersed the API and MgSt uniformly, which in turn enabled full dissolution. 

The Turbuhaler^®^ can deliver 1 mg of soft agglomerates per suction, which is sufficient for most inhaled corticosteroids [60,61]. Further work should focus on the investigation of different proportions of drug and lactose to achieve the required dose strength for different indications. Different types of ternary components can also be applied for mechanical dry-coating to reduce the interparticulate interactions. This could optimize the agglomerate formulation system used with the Turbuhaler^®^ to maximize the aerosolization and mitigate its side effects in clinical trials. 

## 5. Conclusions

This study demonstrated that the production of soft agglomerate particles from fine API and lactose through the combination of ultrasonic vibration and mechanical dry-coating is a suitable method for producing an easy-to-use powder inhalation formulation with improved aerosol performance and reduced upper airway deposition.

In the standard production process of agglomerate formulations, a classifying step is carried out, in which unwanted particle fractions with either higher mechanical strength or finer particles with poor flowability are removed. This inevitably limits the batch size during production. In addition, the limited capacity of the rotation pan and compression from the self-weight of the overlying powder also create problems for process scale-up. The combination of ultrasonic vibration and mechanical dry-coating, producing a uniform agglomerate size distribution without a classification step, enables the continuous production of agglomerate formulations for industrial application. Moreover, the agglomerate formulations illustrated improved aerosolization, which was represented by a higher FPF and a smaller artificial throat deposition fraction, as measured using NGI testing, and superior dispersion, which was characterized using real-time monitoring of the fine particle release profiles. Such improvements in aerosol performance were attributed to the reductions in the rupture force of agglomerate particles and the intrinsic powder cohesion after surface modification. 

The significant reduction in drug particle deposition in the upper airway and improved aerosolization indicated the advantages provided by agglomerate formulations produced using this combined production method. The proposed method may have further value in the development of other inhaled corticosteroid powder formulations, providing superior therapeutic effects and less oral cavity infection.

## Figures and Tables

**Table 1:** Particle size and SSA of powder bulk samples.

Formulation Composition	Particle Size (µm)			SSA (m^2^/g)
	D_10_	D_50_	D_90_	
Budesonide	0.51 ± 0.01	1.56 ± 0.07	3.23 ± 0.15	6.15 ± 0.12
MgSt	0.50 ± 0.01	1.43 ± 0.04	3.07 ± 0.05	8.66 ± 0.15
Lactose LH300	1.08 ± 0.06	2.99 ± 0.99	6.97 ± 0.62	3.92 ± 0.48
Lactose–0.5% MgSt	0.62 ± 0.01	2.41 ± 0.03	7.38 ± 0.03	2.80 ± 0.43
Lactose–1% MgSt	0.56 ± 0.02	2.11 ± 0.04	6.93 ± 0.07	2.35 ± 0.11
Lactose–3% MgSt	0.50 ± 0.00	1.96 ± 0.03	6.92 ± 0.04	2.62 ± 0.07

**Table 2:** Elemental analysis (EA) of the mixture after mechanical dry-coating with EDX.

MgSt	Atom Type	C	O	Mg
0.5%	* MoF, sto	50.45	49.53	0.02
	MoF, EDX	46.66 ± 0.20	53.31 ± 0.39	0.03 ± 0.01
1%	MoF, sto	50.63	49.32	0.04
	MoF, EDX	47.16 ± 0.20	52.79 ± 0.39	0.05 ± 0.01
3%	MoF, sto	51.84	48.04	0.12
	MoF, EDX	48.72 ± 0.20	51.13 ± 0.38	0.15 ± 0.01

* Molar fractions (MoFs) are expressed in % and were calculated from stoichiometry (sto) and EDX.

**Table 3:** Overview results of bulk properties for the mixture (*n* = 3).

Mixture	M1-0%	M2-0.5%	M3-1%	M4-3%
Drug content (%)	99.0 ± 0.6	99.4 ± 0.5	100.6 ± 1.2	104.3 ± 1.5
Bulk density (g/mL)	0.22 ± 0.01	0.26 ± 0.01	0.26 ± 0.03	0.25 ± 0.01
Tapped density (g/mL)	0.35 ± 0.01	0.45 ± 0.02	0.45 ± 0.02	0.46 ± 0.02
Total surface energy, $\gamma_{s}^{T}$ (mJ/m^2^)	315.0 ± 8.0	253.0 ± 6.3	125.9 ± 2.9	99.3 ± 2.1

**Table 4:** Properties of soft agglomerates under various ultrasonic powers (*n* = 3).

Power/Watts	F1-0 W	F2-100 W	F3-200 W	F4-400 W
$X_{EQPC}$ (mean ± SD)/µm	563.8 ± 79.4	552.2 ± 75.6	424.3 ± 46.3	379.6 ± 88.3
Sphericity (mean ± SD)	0.8 ± 0.0	0.9 ± 0.0	0.9 ± 0.0	0.9 ± 0.0
Drug content/%	81.6 ± 1.0	95.0 ± 2.2	98.9 ± 0.6	98.6 ± 3.1
Rupture force/mN	5.3 ± 0.5	4.5 ± 0.8	3.6 ± 1.6	0.8 ± 0.2
Rupture Deformation (%)	4.3 ±1.2	5.2 ± 1.1	4.7 ± 0.9	3.6 ± 1.1

**Table 5:** Aerodynamic properties of the formulation prepared with different vibration intensities (*n* = 3).

	F1-0 W	F2-100 W	F3-200 W	F4-400 W
Budesonide				
ED/%	78.7 ± 4.6	81.6 ± 2.3	82.2 ± 1.9	82.7 ± 1.7
FPD/µg	57.0 ± 6.2	73.5 ± 10.4	80.3 ± 6.6	88.8 ± 5.2
FPF/%	36.2 ± 3.0	45.0 ± 5.6	48.8 ± 2.8	53.2 ± 4.1
MMAD/µm	2.7 ± 0.1	2.6 ± 0.1	2.5 ± 0.0	2.4 ± 0.2
GSD	1.8 ± 0.0	1.7 ± 0.0	1.7 ± 0.1	1.8 ± 0.1
Lactose				
ED/%	64.5 ± 6.7	78.8 ± 2.3	86.0 ± 2.9	84.5 ± 4.0
FPD/µg	164.1 ± 27.4	230.4 ± 13.8	306.1 ± 12.1	355.1 ± 18.2
FPF/%	31.8 ± 4.1	36.6 ± 1.3	45.2 ± 0.8	52.5 ± 4.6
MMAD/µm	2.7 ± 0.1	2.6 ± 0.1	2.5 ± 0.0	2.4 ± 0.2
GSD	1.8 ± 0.0	1.7 ± 0.0	1.7 ± 0.1	1.8 ± 0.1

**Table 6:** Agglomerates bulk properties and aerodynamic performance (*n* = 3) for formulation with different preparation methods.

	Spheroidization without Ultrasonic Vibration			Spheroidization with Ultrasonic Vibration		
	F5-0.5% MgSt	F6-1% MgSt	F7-3% MgSt	F8-0.5% MgSt	F9-1% MgSt	F10-3% MgSt
$X_{EQPC}$/μm	489.7 ± 86.6	452.6 ± 75.5	389.0 ± 61.3	428.0 ± 34.3	408.4 ± 26.1	364.7 ± 23.7
Ruptureforce/mN	4.5 ± 0.8	3.9 ± 0.7	3.6 ± 0.6	2.6 ± 0.4	2.2 ± 0.2	1.8 ± 0.1
Drug content/%	87.8 ± 1.2	96.2 ± 0.9	97.7 ± 0.2	98.6 ± 3.1	101.3 ± 1.3	98.6 ± 3.1
ED/%	83.5 ± 6.3	90.2 ± 5.9	93.4 ± 0.9	93.5 ± 1.8	92.9 ± 5.3	95.8 ± 3.9
FPD/μg	79.3 ± 7.1	92.3 ± 11.0	102.9 ± 13.9	118.7 ± 11.6	132.0 ± 5.2	136.7 ± 5.1
FPF/%	47.2 ± 5.8	51.4 ± 4.2	55.1 ± 6.9	63.4 ± 5.1	71.1 ± 1.3	71.4 ± 1.7
MMAD/μm	2.5 ± 0.2	2.5 ± 0.2	2.3 ± 0.3	2.2 ± 0.1	2.2 ± 0.2	2.1 ± 0.2
GSD	1.8 ± 0.0	1.8 ± 0.1	1.7 ±0.1	1.7 ± 0.0	1.7 ± 0.0	1.7 ± 0.0

ED = emitted dose; FPD = fine particle dose; FPF = fine particle fraction; GSD = geometric standard deviation; MMAD = mass median aerodynamic diameter; data are presented as mean ± SD.

## Data Availability

No new data were created.
